# Transient neonatal zinc deficiency or acrodermatitis enteropathica?^☆^

**DOI:** 10.1016/j.abd.2023.08.018

**Published:** 2024-07-01

**Authors:** Luciane Francisca Fernandes Botelho, Selma Hélène, Carolina Gonçalves Contin Proença, Silvia Assumpção Soutto Mayor

**Affiliations:** Dermatology Clinic, Hospital da Santa Casa de Misericórdia de São Paulo, São Paulo, SP, Brazil

*Dear Editor,*

A five-month-old male patient presented with erythematous-desquamative lesions of two months duration, initially in the perioral region, which progressed to the following regions: acral, perinasal, genital, shoulders and occipital with associated alopecia (Fig. 1). There was no diarrhea or systemic involvement. He had been treated with antifungals, topical corticosteroids and systemic antibiotics in another service without improvement. A clinical hypothesis of acrodermatitis enteropathica *versus* transient neonatal zinc deficiency (TNZD) was made and serum zinc and alkaline phosphatase measurements were requested, the results of which showed no alterations. His personal history showed he had been preterm (34 weeks) and exclusively breastfed. Zinc measurement in breast milk could not be performed, as it is not a commercially available test in Brazil.

**Figure 1:**
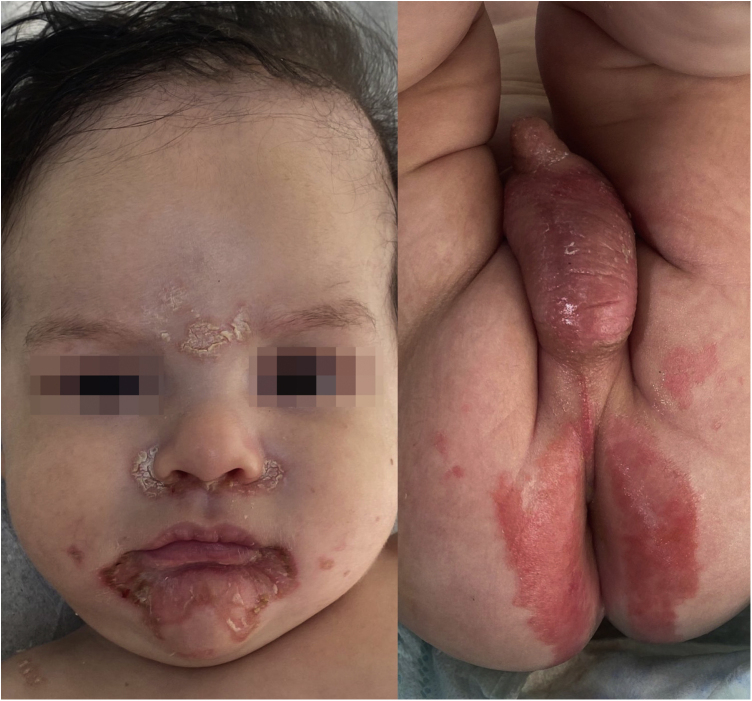
Erythematous-desquamative lesions in the perioral, perinasal, glabella and genital regions.

Given the characteristic clinical picture and the extent of the skin lesions, oral zinc supplementation was started at a dose of 3 mg/kg/day. After three weeks of supplementation, the patient significantly improved (Fig. 2), confirming the diagnosis of zinc deficiency, despite normal serum zinc levels. The patient continued to be monitored and at the age of one year and three months, complete exome sequencing was performed, which identified a mutation in the SLC30A2 gene. The mother did not undergo the genetic test, but the patient probably inherited the mutation from the mother who produced milk with a low concentration of zinc. The authors concluded that it was a case of TNZD and supplementation was suspended. The patient showed no recurrence of lesions.

**Figure 2:**
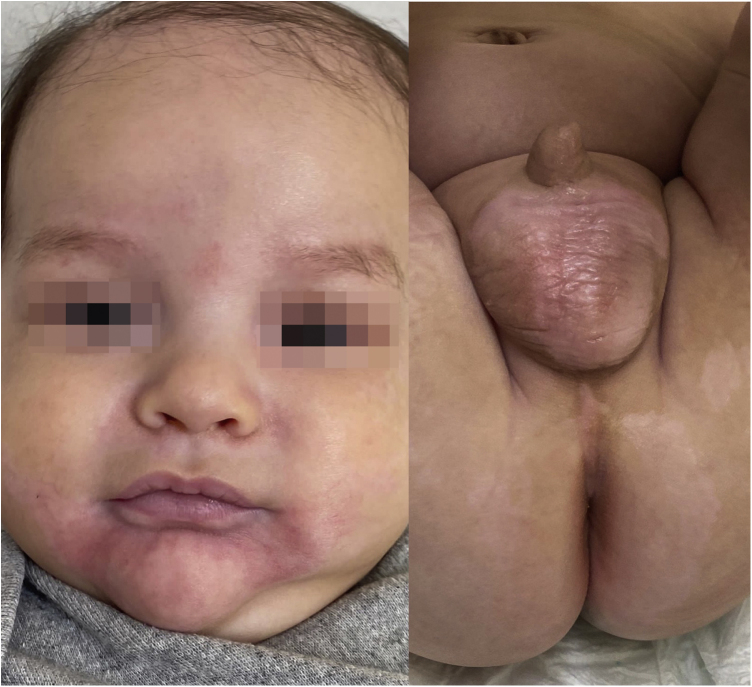
Three weeks after zinc supplementation, the patient showed significant improvement.

Zinc acts on cell development, differentiation and growth.^1^

Zinc deficiency can be either genetic or acquired. The genetic forms are autosomal recessive, known classically as acrodermatitis enteropathica, or autosomal dominant called TNZD.^2^ In acrodermatitis enteropathica, the mutation occurs in the SLC39A4 gene, and the incidence of this mutation is estimated at 1/500,000 children without predilection for gender or ethnicity. The SLC39A4 gene encodes the ZIP4 protein, present in intestinal enterocytes, responsible for zinc absorption.^3^ TNZD occurs in children who are exclusively breastfed, due to the low concentration of zinc in breast milk, resulting from the maternal mutation in the SLC30A2 gene. This gene encodes a zinc transporter called ZNT2 present in mammary gland cells and responsible for transporting zinc into breast milk.^2^ Table 1 depicts the main differences between acrodermatitis enteropathica and TNZD.

**Table 1:** Characteristics of transient neonatal zinc deficiency and acrodermatitis enteropathica.

	Transient neonatal zinc deficiency	Acrodermatitis enteropathica
Genetic mutation and inheritance	SLC30A2, autosomal dominant	SLC39A4, autosomal recessive
Moment of clinical presentation	It occurs in the first months of life when the child is exclusively breastfeeding	It usually occurs after breastfeeding discontinuation
Serum zinc measurement	Reduced, rarely normal	Reduced, rarely normal
Zinc measurement in breast milk	Reduced	Normal
Maternal serum zinc measurement	Normal	Normal
Need for zinc supplementation	Only during exclusive breastfeeding	Throughout life

The acquired forms of zinc deficiency may be due to preterm birth, zinc-deficient parenteral nutrition and intestinal malabsorption syndromes.^3^, ^4^

The clinical picture of the genetic and acquired forms is similar; in both forms, the patients show erythematous-desquamative plaques, and vesicles, bullae and pustules may appear in the periorificial and acral regions. Other manifestations include alopecia, diarrhea, angular cheilitis, paronychia, growth retardation, neurological deficit, difficulty in healing, anemia, photophobia, hypogeusia, anorexia, pubertal delay, and hypogonadism.^3^, ^5^ The symptoms of classic acrodermatitis enteropathica usually appear when breastfeeding is stopped, as breast milk is rich in bioavailable zinc; however, in preterm infants with a high zinc demand, symptoms may appear during breastfeeding.^4^

Among the differential diagnoses, we must consider biotin deficiency, fungal infections, seborrheic dermatitis, psoriasis, Langerhans cell histiocytosis, and contact dermatitis.^6^

The present case was a diagnostic challenge, as serum zinc levels were normal, but according to the literature, there are reports of acrodermatitis enteropathica with normal serum zinc levels.^7^, ^8^, ^9^ This paradox is, in part, a result of the homeostasis of plasma zinc, which comprises only 0.1% of total body zinc reserves. Therefore, accurately measuring zinc levels in the body is difficult. A reduced serum level of alkaline phosphatase, a zinc-dependent metalloenzyme, may help in the diagnosis.^3^

With adequate treatment, changes resulting from zinc deficiency resolve without sequelae, but prolonged periods of deficiency can permanently affect growth and neurological development. Zinc supplementation in children with a transient deficiency should be carried out with 1 mg/kg/day and patients with acrodermatitis enteropathica should receive 3 mg/kg/day of supplementation.^10^ In acrodermatitis enteropathica, the supplementation must be maintained throughout life, whereas in TNZD, the supplementation can be suspended after the period of exclusive breastfeeding.^2^

This case report shows the importance of genetic testing in the diagnosis of zinc deficiency in infants, since regardless of the etiology, the clinical manifestations can be identical.

## Financial support

None declared.

## Authors' contributions

Luciane Francisca Fernandes Botelho: Design and planning of the study; drafting and editing of the manuscript; data survey, collection, analysis and interpretation of data; critical review of the literature; approval of the final version of the manuscript.

Selma Hélène: Analysis and interpretation of data; intellectual participation in the propaedeutic and/or therapeutic conduct of the studied case; critical review of the literature; approval of the final version of the manuscript.

Carolina Gonçalves Contin Proença: Intellectual participation in the propaedeutic and/or therapeutic conduct of the studied case; critical review of the literature; critical review of the manuscript; approval of the final version of the manuscript.

Silvia Assumpção Soutto Mayor: Design and planning of the study; analysis and interpretation of data; intellectual participation in the propaedeutic and/or therapeutic conduct of the studied case; critical review of the literature; critical review of the manuscript; approval of the final version of the manuscript.

## Conflicts of interest

None declared.
